# Antibiotic prescription among children with common cold at a district hospital in Uganda

**DOI:** 10.4102/phcfm.v15i1.4106

**Published:** 2023-07-31

**Authors:** Brenda Tusubira, Lillian N. Mukisa, Vicent Okuuny, Innocent Besigye

**Affiliations:** 1 Department of Family Medicine, College of Health Sciences, Makerere University, Kampala, Uganda; 2 Medical Department The Surgery Hospital, Kampala, Uganda

**Keywords:** inappropriate antibiotic prescription, children, respiratory tract infections, Uganda

## Abstract

**Background:**

Most childhood infections are of viral origin making antibiotics unnecessary. They are, however, the most frequently prescribed drugs dispensed to children, resulting in inappropriate antibiotic prescriptions, which are one of the main drivers of antibiotic resistance.

**Aim:**

The study aimed to determine the prevalence of antibiotic prescriptions and identify its associated factors among children below 5 years with common cold who attend the outpatient department in Tororo General Hospital.

**Setting:**

The study was carried out in Tororo General Hospital, Eastern Uganda.

**Methods:**

A cross-sectional survey using consecutive sampling was performed among children below 5 years with common cold attending the outpatient department. Data were collected using an interviewer-administered, structured questionnaire and analysed using STATA version 14.0. Prevalence of antibiotic prescriptions was calculated. Bivariate analysis using chi-square test and multivariate analysis using logistic regression was performed to establish factors associated with antibiotic prescription.

**Results:**

The prevalence of antibiotic prescriptions for common cold among children below 5 years was 23.3%. Factors associated with antibiotic prescription for common cold were duration of symptoms of more than 5 days (OR, 95% CI: 4.49; 1.16–17.23, *p* = 0.029) and being attended to by a clinical officer (OR, 95% CI: 0.19; 0.04–0.91, *p* = 0.038).

**Conclusion:**

There is inappropriate antibiotic prescription among children with common cold in Tororo General Hospital. There is need for antibiotic stewardship programmes to promote optimal antibiotic use in primary care facilities.

**Contribution:**

The study’s findings can be used to develop context-specific antibiotic stewardship programmes tailored to promote judicious use of antibiotics in primary care.

## Introduction

Antibiotics are the most frequently prescribed drugs dispensed to children.^1^ In Uganda, 59.1% of antibiotic prescriptions are given to children below 5 years,^2^ yet most of their infections are of viral origin and self-limiting. Therefore, treatment with antibiotics is unnecessary in most cases.^3^

The unnecessary use of antibiotics is one of the main drivers of antibiotic resistance.^4^ Antibiotic resistance is now a global concern with significant threat to human health.^5^ In 2019, 4.95 million deaths were associated with bacterial antimicrobial resistance globally; 1.27 million of these deaths were directly attributable to antimicrobial resistance.^6^ Despite this, there are few prospects for the development of new classes of antibiotics in the short term. This impacts the ability to treat infectious diseases because of high costs and low availability of alternative treatments especially in low- and middle-income countries, which have the majority of infectious diseases burden.^5,7,8^

A situational analysis on antimicrobial resistance performed by the Uganda National Academy of Science in 2015 showed increasing resistance to commonly used antibiotics with some as high as 80%.^9^ In a cross-sectional study performed on susceptibility patterns in individuals visiting outpatient clinics in urban and rural areas in Uganda, high rates of antibiotic resistance were detected among *Escherichia coli* and *Klebsiella pneumoniae* isolates.^10^ At Mulago National Referral Hospital, a considerable number of pathogens isolated from bloodstream infections demonstrated antimicrobial resistance to first-line drugs.^11^

Primary care accounts for 80% of antibiotic use.^3,12^ For the majority of the population, this is the first point of contact for healthcare. Most prescribing therefore happens at this point.^13^ The most prevalent indication for prescribing antibiotics in primary care is respiratory tract infection (RTI).^3,14^ Eighty percent of children below the age of 5 years diagnosed with respiratory illness in low- and middle-income countries are prescribed an antibiotic despite the majority being of viral origin.^2,15^

Studies in Uganda have found RTIs to be inappropriately managed among children. In Kampala, RTIs were inappropriately managed with antibiotics mostly being used for treatment of common cold.^16^ A household survey in Northern Uganda found 60.2% of children with acute RTIs were prescribed antibiotics.^17^ In South Western Uganda, children diagnosed with RTI at level III and IV health centres received the most antibiotic prescriptions.^18^ Little is known about antibiotic prescription for RTI among children attending primary care facilities in Eastern Uganda. This study, therefore, sought to determine the prevalence and factors associated with antibiotic prescriptions for children below 5 years of age with common cold who attend the outpatients’ department in Tororo General Hospital (TGH), a primary care facility in Eastern Uganda.

## Research methods and design

### Study design

A cross-sectional survey was performed using quantitative methods.

### Study setting

The study was performed in the paediatric section of the outpatients’ department of TGH, a primary care facility. It is a government-owned hospital operating at 224 bed capacity with both inpatient and outpatient services. The hospital is located in Tororo District in Eastern Uganda that has a population of 517 080. Languages spoken are English, Ateso and Japadhola.^19,20^ The outpatient department runs daily from Monday to Friday and is run by clinical officers with nurses sometimes being involved in patient consultations. The patients are first seen by clinical officers and nurses. The medical officers are called in occasionally for consults or to review cases that are challenging or in diagnostic uncertainty. In TGH, exercise books are used as patient files in the outpatient department. These are kept by the patient who returns with the same book for each visit to the hospital. An average of 633 children below the age of 5 years are seen every month in the paediatric section of the outpatient department. A total of 2117 children with common cold were seen in the paediatric section of the outpatient department of TGH from July 2017 to June 2018. The estimated number seen per month with common cold was 177.

### Study population

The study population was children below the age of 5 years with common cold who attended the paediatric section of the outpatient department at TGH within the study period.

### Sample size

A sample size of 382 was used in this study. This was calculated using the Kish-Leslie formula for cross-sectional studies.^21^ The parameters used in sample size estimation were a 95% confidence level (CI), a 5% level of precision and a proportion of inappropriate antibiotic use of 53.5%.^16^

### Sampling procedure

Consecutive sampling was used. Every child that presented to the outpatient department and fit the inclusion criteria during the study period was included in the study.

### Inclusion and exclusion criteria

The inclusion criteria were children below the age of 5 years with common cold who attended the outpatient department of TGH. Children who were diagnosed with other conditions that according to the Uganda Clinical Guidelines (UCG) required antibiotic treatment, and those with caregivers less than 18 years of age were excluded from the study.

### Data collection

Data were collected from 9 September 2020 to 30 November 2020 using a pretested, interviewer-administered, structured questionnaire. In the outpatient department at triage, children under the age of 5 years with common cold were identified by research assistants. The research assistants were clinical officers who had been trained in identification of children with common cold. During the study period, the research assistants were not involved in patient consultation. In this study, the common cold was defined according to the UCG and the classification of ‘no pneumonia; cough or cold’ in the Integrated Management of Childhood Illness.^22,23^ After completing the consultation with a health professional, an exit interview with the child’s caregiver was performed by the research assistants in English or the local languages as preferred by the caregiver. Information on patient’s age, sex, presence of a fever and duration of symptoms was obtained from the caregiver. From the patient’s book, information on whether laboratory investigations were performed, whether antibiotics had been prescribed or not and prescriber were obtained and fed into the questionnaire. A sticker placed at the back of the child’s file allowed for identification so that the same child was not included more than once in the study.

Data collected were periodically checked by the principal investigator. Any questionnaires found to have inaccurate or inconsistent data were discarded and data collection continued until the required sample size of 382 was obtained. A total of 391 participants were interviewed; 9 of the questionnaires had incomplete information and were therefore discarded.

For each of the staff working in the paediatric section of the outpatient department, a code was assigned prior to onset of data collection. This code was filled into the questionnaire to represent the health professional cadre that the patient consulted. After data collection was completed, with required sample size obtained, information on cadre and number of years in practice was found out from the healthcare professionals working in the paediatric section of the outpatient department. This information was then entered into the questionnaires using the codes to identify the health professional cadre that consulted with patients.

### Data analysis

Data were entered into EPIDATA version 3.1 and were then exported to STATA version 14.0 for analysis. Descriptive statistics were used to summarise the patient, health facility and healthcare provider characteristics. The proportion of children with common cold who were prescribed antibiotics was calculated. The proportions of children who received antibiotic prescriptions for common cold under the different patient, health provider and health facility characteristics were compared using chi-square test at 95% CI (*p* < 0.05). A logistic regression model was used to establish factors that were associated with antibiotic prescription in children with common cold. Variables whose *p*-value of the unadjusted odds ratio (OR) at bivariate level was less than 0.2 were considered at multivariate analysis.

### Ethical considerations

Permission to do the study was sought from Makerere University’s Department of Family Medicine College of Health Sciences. Ethical approval was obtained from the School of Medicine Research and Ethics Committee, Makerere University College of Health Sciences (No. REC REF 2020-116). Permission to carry out the study was obtained from the Office of the Hospital Superintendent, TGH.

Informed consent was obtained from the caregivers of the children eligible for the study and from the healthcare professionals prior to asking them about their cadre and years of experience. Privacy and confidentiality of participants were observed by de-identification of data.

## Results

A total of 382 children were included in this study. There were 180 (47.1%) males and 202 (52.9%) females with a mean age of 28.3 (+13.0) months and median age of 26 (21–36) months. The mean duration of symptoms was 3 days. Table 1 shows the patient, health provider and health facility characteristics.

**TABLE 1:** Patient, health provider and health facility characteristics (*N* = 382).

Characteristics	Frequency	Percentage
**Gender**		
Male	180	47.1
Female	202	52.9
**Age in completed months**		
0–23	104	27.2
24–59	278	72.8
**Fever**		
No fever	41	10.7
Fever	341	89.3
**Duration of symptoms (days)**		
1–5	371	97.1
> 5	11	2.9
**Experience of healthcare professional (years)**		
0–10	123	32.2
> 10	259	67.8
**Cadre of healthcare professional**		
Medical officer	7	1.8
Clinical officer	347	90.8
Nurse	21	5.5
Student (DCM)	7	1.8
**Laboratory investigations**		
No	87	22.8
Yes	295	77.2

DCM; Diploma Clinical Medicine.

The prevalence of antibiotic prescription in children with common cold below the age of 5 years who attend the outpatient department in TGH was 23.3%.

Table 2 shows the proportions of children who were prescribed antibiotics within the various patient characteristics. The proportion of children who received antibiotic prescriptions was observed to be higher in males, 24 months or older, those with no fever and those who had symptoms for more than 5 days.

**TABLE 2:** Proportion of children who were prescribed antibiotics within the different patient characteristics (*N* = 382).

Patient characteristics	*n*	Proportion prescribed antibiotics (%)	Proportion not prescribed antibiotics (%)
**Gender**			
Male	180	24.0	76.0
Female	202	23.0	77.0
**Age in completed months**			
0–23	104	17.3	82.7
24–59	278	25.5	74.5
**Fever**			
No fever	41	39.0	61.0
Fever	341	21.4	78.6
**Duration of symptoms (days)**			
1–5	371	22.4	77.6
> 5	11	54.5	45.5

**FIGURE 1:**
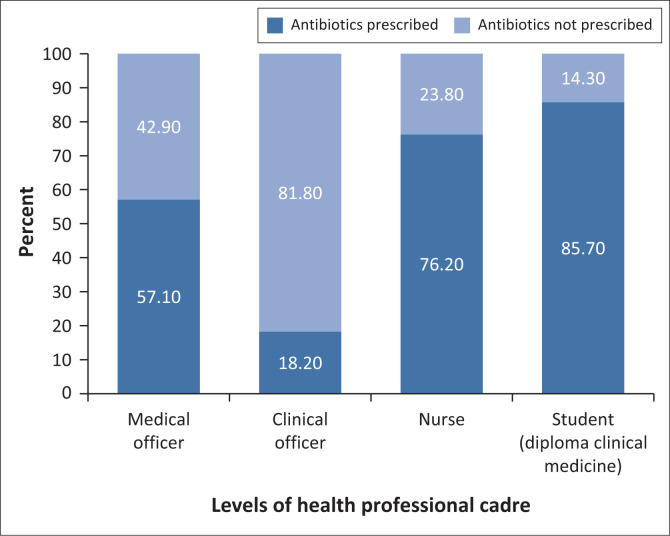
Proportion of children who were prescribed antibiotics among different levels of health professional cadre.

Proportion of antibiotic prescriptions among the different levels of health professional cadre is shown in Figure 1. The highest proportion of antibiotic prescriptions was found in children seen by a student and lowest in those seen by a clinical officer.

The proportion of children who were prescribed antibiotics was higher among children who did not have any laboratory investigations performed, with 32.2% being prescribed antibiotics. Among those who had laboratory investigations carried out, 20.7% were prescribed antibiotics. Among the children who were attended to by a health professional with 0–10 years of experience, 37.4% were prescribed antibiotics. Of the children who were attended to by a health professional with more than 10 years of experience, 16.6% were prescribed antibiotics.

The proportions of children who received antibiotic prescriptions for common cold under the different patient, health provider and health facility characteristics were compared using chi-square test at 95% CI (*p* < 0.05). Table 3 shows the comparative results.

**TABLE 3:** Factors associated with antibiotic prescription in children below the age of 5 years with common cold (*N* = 382).

Factor	Antibiotics prescribed				*n*	%	*p*-value
	Yes		No				
	89	23.3%	293	76.7%
**Gender of child**							0.797†
Male	43	24.0	137	76.0	180	47.1	-
Female	46	23.0	156	77.0	202	52.9	-
**Child age in completed months**							0.090†
0–23	18	17.3	86	82.7	104	27.2	-
24–59	71	25.5	207	74.5	278	72.8	-
**Does the child have a fever**							0.012†
No fever	16	39.0	25	61.0	41	10.7	-
Fever	73	21.4	268	78.6	341	71.7	-
**Duration of symptoms in days**							0.013†
1–5	83	22.4	288	77.6	371	97.1	-
> 5	6	54.5	5	45.5	11	2.9	-
**Experience of healthcare professional (years)**							< 0.001†
0–10	46	37.4	46	37.4	123	32.2	-
> 10	43	16.6	43	16.6	259	67.8	-
**Laboratory investigations**							0.026†
Yes	61	20.7	77	62.6	295	77.2	-
No	28	32.2	216	83.4	87	22.8	-
**Cadre of healthcare professional**							< 0.001‡
Medical officer	4	57.1	3	42.9	7	1.8	-
Clinical officer	63	18.2	284	81.8	347	90.8	-
Nurse	16	76.2	5	23.8	21	5.5	-
Student (DCM)	6	85.7	1	14.3	7	1.8	-

DCM; Diploma Clinical Medicine.

† , Comparisons performed using Pearson’s chi-square test for independent proportions;

‡ , Comparisons performed using Fisher’s exact chi-square test for independent proportions.

Table 4 shows the factors associated with antibiotic prescription in children below the age of 5 years with common cold using bivariate and multivariate logistic regression. Factors that were found to be significant at bivariate level were fitted into the final model and adjustments were performed to control for confounding.

**TABLE 4:** Factors associated with antibiotic prescription in children below the age of 5 years with common cold.

Characteristic	Unadjusted OR	95% CI	*p*-value	Adjusted OR	95% CI	*p-*value
**Gender**						
Male	1.00	-	-	-	-	-
Female	0.94	0.58–1.58	0.797	-	-	-
**Age in completed months**						
0–23 months	1.00	-	-	1.00	-	-
24–59 months	1.64	0.92–2.91	0.092	1.57	0.83–2.96	0.168
**Duration of symptoms (days)**						
1–5	1.00	-	-	1.00	-	-
> 5	4.16	1.24–14.0	0.021	4.49	1.16–17.53	**0.029**
**Fever**						
No fever	1.00	-	-	1.00	-	-
Fever	0.43	0.22–0.84	0.014	0.61	0.29–1.34	0.218
**Laboratory investigations**						
Yes	1.00	-	-	1.00	-	-
No	1.82	1.07–3.10	0.027	1.50	0.81–2.77	0.201
**Level of cadre of healthcare professional**						
Medical officer	1.00	-	-	1.00	-	-
Clinical officer	0.17	0.04–0.76	0.021	0.19	0.04–0.91	0.038
Nurse	2.40	0.40–14.56	0.341	1.76	0.25–12.38	0.565
Student (DCM)	4.50	0.34–60.15	0.256	3.02	0.20–44.90	0.423
**Experience of healthcare professional (years)**						
0–10	1.00	-	-	1.00	-	-
> 10	0.33	0.20–0.54	< 0.001	0.56	0.31–1.01	0.052

Note: Bold values are *p*-values that are less than 0.05 and highlight the characteristics that were significant at multivariate analysis after adjustment to control for confounding.

OR; odds ratio, CI; confidence interval, DCM; Diploma in Clinical Medicine.

The following characteristics were significant before adjustment: (1) duration of symptoms of more than 5 days, (2) presence of a fever, (3) experience of healthcare professional, (4) being attended to by a clinical officer and (5) doing laboratory investigations.

After adjustment, duration of symptoms was significantly associated with antibiotic prescription for common cold. Children with duration of symptoms of more than 5 days were at least four times more likely to receive an antibiotic prescription for common cold compared with those with symptoms for 5 days or less.

A child being seen by a clinical officer was also found to be significantly associated with antibiotic prescription for common cold. Children with common cold who were attended to by a clinical officer were 81% less likely to receive an antibiotic prescription compared with those seen by a medical officer.

There was no significant association between the following factors and antibiotic prescription in children with common cold below the age of 5 years: age of child, fever, experience of healthcare professional, whether laboratory investigations were performed or not.

## Discussion

In this study, antibiotics were prescribed to 23.3% of children who presented with common cold despite the fact that the common cold is a self-limiting viral infection and antibiotics play no role in management.^24,25,26^ Many healthcare providers think viral infections can be treated with antibiotics.^27^ In Buyende district, Eastern Uganda, 64% of health workers recommended use of antibiotics for treatment of common cold.^28^ In Papua New Guinea, despite 95% of the health workers surveyed knowing viruses were the cause of common cold, 30% thought antibiotics were needed.^29^ There also seems to be a gap between knowledge and practice. In Shandong province, China, despite 87% of doctors reporting they would refuse to prescribe antibiotics for common cold, 55% of patients diagnosed with common cold were prescribed an antibiotic.^30^

The prevalence of antibiotics for children in TGH was lower than that found in other studies. In Gambia, 51.74% of children with common cold were prescribed antibiotics and 82.4% were prescribed antibiotics in Papua New Guinea.^29,31^ In Uganda, studies have found a higher prevalence of antibiotic prescription for RTI compared with that found at TGH.^16,17,18^ This study was done in one health facility and the focus was on common cold. The other studies looked at all RTIs with household surveys being performed in Gulu and Kampala and more than one health facility being part of the study in Mbarara. The prevalence of antibiotic prescriptions in TGH is however higher than that found in Lao People’s Democratic Republic where antibiotics were prescribed for 4.9% of children below the age of 5 years with common cold. The low prevalence was attributed to government interventions concerning inappropriate antibiotic prescriptions, which resulted in a drop from 40% to 4.9%.^32^

In a study carried out in Northern Tanzania, it was observed that lower cadres of health professionals were more likely to prescribe antibiotics when not indicated.^33^ This was not the case at TGH where children who were attended to by a clinical officer were 81% less likely to receive an antibiotic prescription compared with those seen by a medical officer. Low adherence to standard treatment guidelines among physicians has been reported in several studies. A study performed in Sweden reported that none of the physicians in their study prescribed antibiotics strictly in line with current recommendation. In Ghana, the adherence to standard treatment guidelines was 64.5%.^34,35,36^ Lower cadres of health professionals on the other hand tend to prescribe in line with standard treatment guidelines compared with higher cadres of healthcare professionals.^37^ This may explain the reduced likelihood of antibiotic prescriptions seen with clinical officers.

The majority of children in this study were seen by clinical officers. The outpatient department of TGH is managed by clinical officers who are assisted from time to time by nurses. The medical officer is called in to review challenging cases or cases with diagnostic uncertainty. Reasons given for antibiotic prescriptions in other studies include a child that looks unwell and being on the safe side if there is uncertainty about whether the cause of illness is viral or bacterial.^38,39^ This is made worse in the absence of diagnostic support.^8^ In this study, 77.2% of children seen had undergone laboratory investigations. These included either a blood slide for malaria or a malaria rapid diagnostic test, neither of which are helpful in supporting or ruling out bacterial or viral aetiology. Diagnosis is therefore made on clinical basis. This may be the case with children reviewed by the medical officer and the resulting antibiotic prescriptions observed in the absence of appropriate diagnostic support.

Symptoms for more than 5 days were one of the reasons given by primary paediatricians for antibiotic prescriptions.^38^ In this study, children with duration of symptoms of more than 5 days were at least four times more likely to receive an antibiotic prescription for common cold compared with those with symptoms for 5 days or less. This is in agreement with other studies performed elsewhere. Rogawski et al. found that respiratory illnesses were significantly more likely to be treated with antibiotics if the illness was of longer duration. In Papua New Guinea, children with symptoms for 7 or more days were more likely to be prescribed an antibiotic.^29,40^ It is possible that with a longer duration of symptoms, the healthcare provider may start to think of bacterial causes as an explanation for presenting symptoms. This is however not the case. A systematic review on duration of symptoms of RTIs found that in 90% of children, common cold resolved by 15 days.^41^ Therefore, there is no benefit obtained from treatment with antibiotics even when initiation of treatment is delayed.^42^

### Limitations

The sampling method used was consecutive sampling. It being a non-probability type of sampling may have created selection bias with individuals selected not being fully representative of the entire population. The study was carried out in only TGH, which might have created selection bias and limited external validity; therefore, findings cannot be generalised.

## Conclusions and recommendations

There is inappropriate prescription of antibiotics among children below the age of 5 years with common cold attending the outpatient department at TGH. Duration of symptoms of more than 5 days and a child being attended to by a clinical officer are significantly associated with antibiotic prescription.

Further studies are recommended to explore the reasons behind inappropriate antibiotic prescriptions among healthcare workers, the prescription patterns among different cadres of health professionals and appropriate diagnostic support. Similar studies carried out in other primary care health facilities will build an information base geared towards forming evidence-based interventions that will help in controlling the spread of antimicrobial resistance.

There is a need for antibiotic stewardship programmes to promote optimal use of antibiotics. This involves the training of healthcare workers in the management of common childhood illnesses, appropriate prescription of antibiotics and antibiotic resistance. Initial training should be followed up with continuous medical education to ensure up-to-date information and supported by periodic clinical audits to enable a positive practical learning environment and improve quality of patient care.
